# Analysis of the genetic basis of periodic fever with aphthous stomatitis, pharyngitis, and cervical adenitis (PFAPA) syndrome

**DOI:** 10.1038/srep10200

**Published:** 2015-05-19

**Authors:** Silvio Alessandro Di Gioia, Nicola Bedoni, Annette von Scheven-Gête, Federica Vanoni, Andrea Superti-Furga, Michaël Hofer, Carlo Rivolta

**Affiliations:** 1 Department of Medical Genetics, University of Lausanne, Lausanne, Switzerland; 2 Pediatric Rheumatology Unit of Western Switzerland, Department of Pediatrics, CHUV, University Hospital of Lausanne, Lausanne, Switzerland; 3 Department of Pediatrics, CHUV, University Hospital of Lausanne, Lausanne, Switzerland; 4 Department of Pediatrics, HUG, Geneva, Switzerland

## Abstract

PFAPA syndrome is the most common autoinflammatory syndrome in children from Western countries. In spite of its strong familial clustering, its genetic basis and inheritance pattern are still unknown. We performed a comprehensive genetic study on 68 individuals from 14 families. Linkage analysis suggested a susceptibility locus on chromosome 8, but direct molecular sequencing did not support this initial statistical finding. Exome sequencing revealed the absence of any gene that was mutated in all patients. Exhaustive screening of genes involved in other autoinflammatory syndromes or encoding components of the human inflammasome showed no DNA variants that could be linked to PFAPA molecular pathology. Among these, the previously-reported missense mutation V198M in the *NLRP3* gene was clearly shown not to co-segregate with PFAPA. Our results on this relatively large cohort indicate that PFAPA syndrome is unlikely to be a monogenic condition. Moreover, none of the several genes known to be involved in inflammation or in autoinflammatory disorders seem to be relevant, alone, to its etiology, suggesting that PFAPA results from oligogenic or complex inheritance of variants in multiple disease genes and/or non-genetic factors.

Autoinflammatory syndromes (AIS) are a group of disorders characterized by attacks of inflammation that are not associated with the identification of autoreactive lymphocytes or of an external inflammation-triggering agent^1^. The majority of these syndromes are inherited as Mendelian traits, although a strong environmental influence has also been suggested for some forms^2^. Genes involved in AIS are linked to the inflammatory activation pathway and in particular to the activation of interleukin 1β (IL1β)^3^.

Periodic fever with aphthous stomatitis, pharyngitis, and cervical adenitis (PFAPA) syndrome belongs to the AIS group, although the etiology of this disease is still unknown. Described for the first time by Marshall in 1987^4^, it is characterized by high fever (higher than 39 °C) lasting three to seven days and reoccurring regularly every three to eight weeks, alongside with at least one of these other symptoms: aphthous stomatitis, pharyngitis, or cervical adenitis^5^. It is an early-onset disease (usually before the age of 5 years) and in general it completely resolves before adulthood. However, cases of PFAPA that were persistent after adolescence have also been reported^6^,^7^,^8^,^9^. Patients are asymptomatic between episodes and have no developmental or growth problems. During PFAPA febrile attacks, an increase of IL1β cytokines has been shown both at the gene^10^ and protein levels^11^, suggesting the involvement of IL1β release in the etiology of the disease.

The genetic origins of PFAPA are one of the most debated features of this syndrome. Although it is generally considered a sporadic disease^12^, familial clustering has been observed, suggesting the presence of a possible hereditary component^13^. It is not unusual to ascertain families having more than one member affected with PFAPA^14^,^15^, and positive family history for recurrent fevers can be detected in about half of PFAPA patients^16^. Several studies have investigated a possible involvement of the genes responsible for Familiar Mediterranean Fever (FMF, gene *MEFV*), TNF-Receptor Associated Periodic Syndrome (TRAPS, gene *TNFRSF1A*) Hyper IgD Syndrome (HIDS, gene *MVK*) and Cryopyrin Associated Periodic Syndrome (CAPS, gene *NLRP3*) in PFAPA cohorts^11^,^17^,^18^,^19^,^20^,^21^. An interesting hypothesis is that PFAPA could represent an unspecific, milder form of other AIS for which DNA variants in known AIS genes and present in the general population could be involved. An example is the *NLRP3* variant V198M (or, more precisely, p.V200M, rs121908147), detected in the control population with an allele frequency of ~1%, and reported as a causative mutation for CAPS^22^, as well as to be associated with milder diseases or with other unspecified AIS^23^,^24^,^25^. V198M was also identified in PFAPA patients, suggesting a possible role of *NLRP3* hypomorphic alleles in the pathogenesis of the disease^11^, although a clear genotype-phenotype association is still missing. Recently, a heterozygous deletion of the gene *SPAG7* consequent to a chromosomal translocation was reported in a single individual affected with PFAPA and displaying other syndromic features such as growth retardation, facial dysmorphisms, and dysplastic brachymesophalangy^26^, However, these data could not be replicated and it is currently not clear whether the link between *SPAG7* and PFAPA is causal or coincidental.

In this work, we perform for the first time an unbiased, comprehensive genetic analysis on a collection of families with PFAPA syndrome. Our data indicate that PFAPA is neither a monogenic disease nor a condition that can be clearly associated with DNA changes in other AIS genes. In addition, we demonstrate that the *NLRP3* DNA variant V198M is not associated with this disease.

## Results

### PFAPA syndrome shows an apparent autosomal dominant with incomplete penetrance pattern of inheritance, if considered as a Mendelian disease

**Figure 1:**
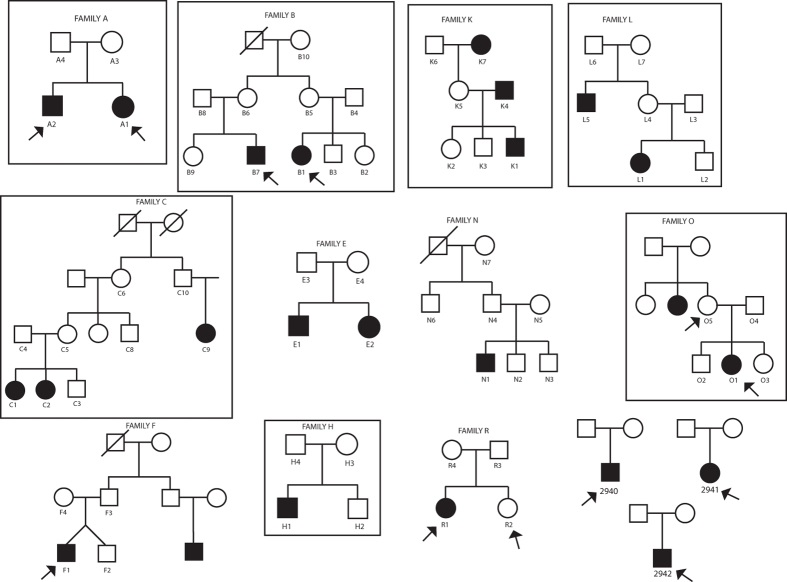
Families analyzed Alphanumeric codes indicate individuals who were investigated by genome-wide SNP genotyping, while arrows indicate individuals whose DNA was processed by exome sequencing (some individuals were analyzed by both techniques). Boxes indicate families that underwent linkage studies.

**Figure 2:**
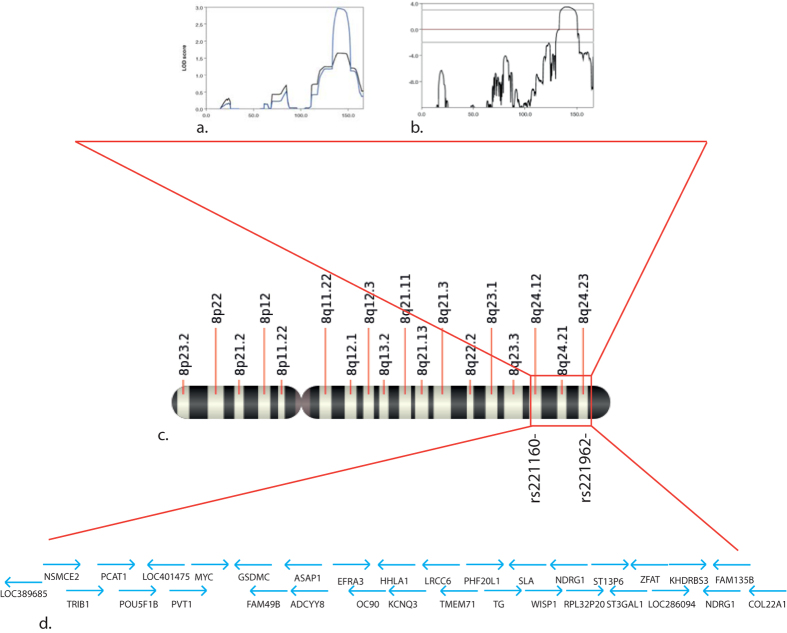
Linkage analysis of PFAPA families **a**) Output of Merlin linkage analysis for linear (black line) and exponential (blue line) non-parametric models. For our study, the exponential model gives a better resolution, since it is indicated for a small number of families and large allelic sharing. **b**) Output for a parametric model (autosomal dominant with incomplete penetrance). The peak at chromosome 8 is still conserved. **c**) Location of the linkage interval on the long arm of chromosome 8, between markers rs221160 and rs221962. **d**) Protein coding genes present in the identified interval.

We analyzed 68 individuals from 14 families segregating PFAPA by genome-wide SNP genotyping, whole-exome sequencing, or both. Pedigree analysis suggested clear inheritance, compatible with an autosomal dominant with incomplete penetrance model, if Mendelian inheritance with no genetic heterogeneity is assumed as a model (Fig. 1). Because of the lack of genetic data for this disease to infer the penetrance factor from the literature, we empirically set it to be around 50% following pedigree examination. Following these observations, we decided to perform a genome-wide linkage analysis to identify a possible locus carrying the causative gene of PFAPA syndrome. We selected a subset of informative families (families A, B, C, H, K, L, O) based on their clinical information. Multifamily whole genome analysis considering non-parametric methods revealed a single and unique peak on chromosome 8 with a maximum LOD score of 2.9 (Fig. 2a). This peak was also conserved with a parametric method by considering a fully penetrant inheritance pattern with a LOD score of 3.4 (Fig. 2b). The identified region was of 11 Mb and spanned bands 8q21.1 to 8q24.4 of chromosome 8 (Fig. 2c). This region is scarcely populated, as it contains only 40 RefSeq genes, of which only 32 were protein coding (Fig. 2d – and Supplementary Table 1). We selected 3 affected individuals (B1, L1 and C2) to be screened for all exons of all genes that are present in the interval. Analysis of DNA variants failed to reveal any single gene that was mutated in all three affected individuals within the identified region, with the exception of two non-coding variants representing common polymorphisms (Supplementary Table 2).

### Exome capture and NGS show no common disease gene associated with PFAPA

Despite the negative results from the screening of the chromosome interval, we could not completely exclude *a priori* a monogenic model of inheritance. Therefore we decided to select some key individuals to be whole-exome sequenced. Inclusion criteria were the availability and the quality of the extracted DNA, as well as the size and the quality of the clinical information regarding the family (Table 1). We sequenced, whenever possible, two affected individuals within the same family, or one obligated carrier and a patient, for a total of 11 disease-carrying subjects and one unaffected family member (Fig. 1, arrows).

Under the hypothesis that PFAPA is a monogenic condition with no genetic heterogeneity, we scanned the list of DNA variants present in all patients, in order to identify a gene carrying rare variants (minor allele frequency, or MAF < 2%) in more than 90% of the analyzed samples (to allow the presence of false negatives). In total, we detected 14 genes that satisfied these criteria (Table 2). Four of them belonged to the *MUC* family and represented false positives results, similar to those obtained by other exome sequencing projects^27^. The same was true for mutations in six other genes (*WNK1*, *ZNF384*, *TTN*, *GOLGAL6L1*, *C11orf40*, and *KIAA1751*) that, for various reasons, could be recognized as sequencing artifacts^28^. The remaining four genes (*NBPF15*, *OR4C45*, *ZNF492*, and *PCMTD1*) had many novel variants, which were however also present in controls. No common gene carrying rare variants that could be compatible with pathogenic mutations could therefore be identified in our cohort.

No rare or potentially abnormal DNA variants were found in the *SPAG7* gene, in any of the patients analyzed.

### Screening for mutations in genes for autoinflammatory recurrent fever conditions does not support their involvement in PFAPA etiology

Since the presence of rare variants in other inflammatory genes could have an impact on the PFAFA phenotype, we decided to search specifically for variants in all known autoinflammatory genes in our cohort (Supplementary Table 3). Screening of the *MEFV* gene, associated with FMF, revealed the presence of polymorphisms in heterozygous state with unknown function for patients O1 (p.I591T, rs11466045, MAF 0.07) and R1 (p.F425Y, rs11466045, MAF 0.001). None of these were present in the other analyzed members of the family. Conversely, in family A, we identified a variant (p.E148Q, rs3732930, MAF 0.06) shared among the two affected individuals. Interestingly, both patients of family A also carried a heterozygous change in *NLRP3* related to CAPS (p.Q703K, rs35829419, MAF 0.04). In family B, we identified a rare variant in the gene *TNFRSF1A*, usually associated with milder forms of TRAPS (p.R121Q, rs4149584, MAF 0.018). This change cosegregated with the disease. We also identified a novel change in the gene *NLRP12*, associated with familial cold autoinflammatory syndrome-2, in patient R1 (p.R211C). This change, located in a well-conserved region of the protein (Table 3), was also present in R1’s unaffected sister.

### The NLRP3 variant p.V198M associated with CAPS does not cosegregate with PFAPA affected status

**Figure 3:**
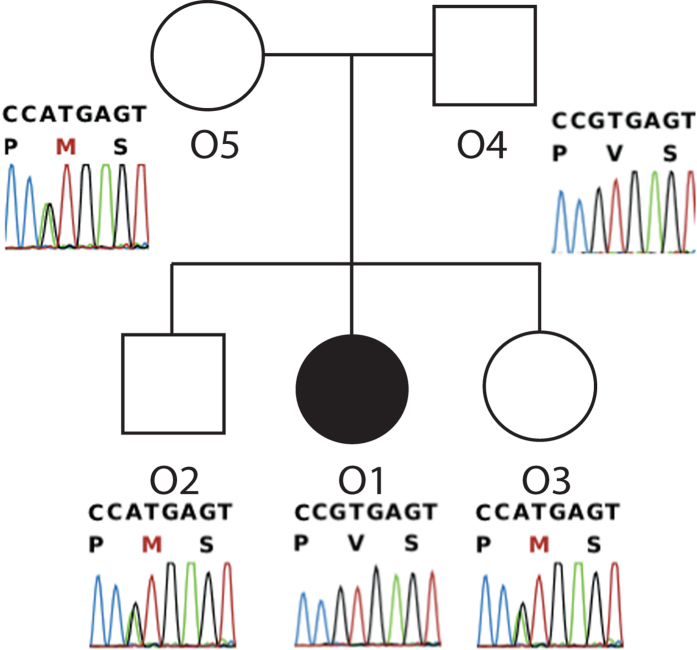
Cosegregation analysis for the V198M variant in *NLRP3*, in family O. Variant V198M (p.V200M) in gene *NLRP3* does not cosegregate with PFAPA in this pedigree (healthy members carry it, whereas the affected member does not), suggesting that this polymorphism is not associated with the disease.

Analysis of *NLRP3* in family O revealed the presence of the variant V198M (p.V200M, rs12908147, MAF 0.01), often associated with milder forms of CAPS^23^,^29^. This variant was found in heterozygous state only in the obligated carrier member O5, whereas it was not present in the proband. Cosegregation analysis on available members of the family revealed that this change is present also in the two healthy siblings and it is absent in the affected one, confirming that this change is not involved in the etiology of PFAPA in this family (Fig. 3).

### Several rare variants with uncertain function were present in inflammasome-related genes

**Figure 4:**
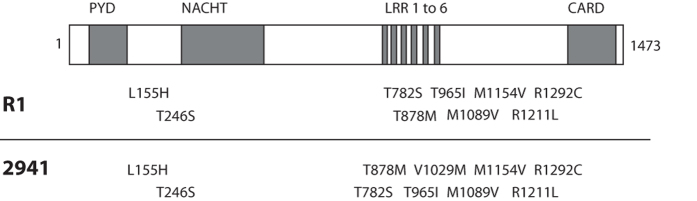
Graphical representation of the haplotype identified on *NLRP1* for two subjects. The gene structure is depicted in the upper part. Amino acid changes identified in these individuals are reported. All these changes have a MAF of <0.1. PYD: pyrin domain; NACHT: Conserved NAIP, CIITA, HET-E and TP-1 domains; LRR: leucine rich receptor domain; CARD: Caspase activator and recruitment domain.

Since an impairment in IL-1β and NF-κB signaling and production has been reported in individuals with PFAPA syndrome^10^, we decided to look closely at all known genes belonging to the inflammasome complex, with particular attention to genes involved in the production of mature IL-1β. All identified variants in these genes are listed in Table 3. We identified two non-annotated changes in *NLRP5,* for patients 2940 and F1 (p.R274Q, and p.N255S, respectively). Again, patient 2940 presented another rare variant in the gene *NLRP2,* p.L607P (rs182098487, MAF 0.001). A novel change, p.G504D, was found in this same gene in patient B7, although it was not found in the affected cousin. Among the Nod-Like Receptor genes, *NLRP2* showed the highest number of variants within PFAPA subjects, but these were also rather common in the control CoLaus Lausanne cohort (p.S4L, rs142463014, for 2492, MAF 0.09; p.I331V, rs61735077, for R1 and R2, MAF 0.05). Another common variant in *NLRP4* was p.T162M (rs117212164, MAF 0.03), co-segregating with the condition in family O and affecting a conserved residue in the NACHT domain of the NLRP4 protein. Among other genes from the NLR families we identified a rare change in *NLRP10*, p.I384T (rs150112481, MAF 0.01) although only in patient O1, and the change p.S1025L (rs11671248, MAF 0.08) in *NLRP11* in patient 2940. Not many rare variants were identified in the coding region of the *NLRP1* gene, with the only exception being p.V939M (rs61754791, MAF 0.01), found only in patient 2942. Interestingly, however, both patient 2941 and family R shared a rare haplotype composed by different missense polymorphisms located at the C-terminal of the protein (Fig. 4). A similar haplotype has been recently associated with an impairment of IL-1β processing by the inflammasome. Common polymorphisms in *CARD8* were found in a heterozygous state in our cohort and these have been previously associated with other defects of the inflammatory system. These polymorphisms were p.C10X (rs2043211, MAF 0.30) found in patients A1, B7, F1, 2940 and p.V148fs (rs140826611, MAF 0.06) in A2, F1, 2941 and 2942. Among other inflammasome genes, we identified a heterozygous missense change in *NLRC4*, better known as IPAF, p.G786V (rs149451729, MAF 0.03) in patient 2941; a novel change in *NOD1* (p.C241G) in patient 2942, again in the NACHT domain of this protein. A summary of all analyzed variants identified in inflammasome genes and their affected functional domain is reported (Table 3).

## Discussion

PFAPA is a common auto-inflammatory disease with an overall good prognosis. Regular PFAPA fever flares have a significant impact on the quality of life of the patients and their families and are interfering with regular school attendance and normal daily activities. The delay to diagnosis may represent several months or years and children may be exposed to unnecessary diagnostic procedures. A better understanding of the pathogenesis of this disease would lead to more specific diagnostic tools and might relieve the suffering of patients and their families. Clearly the pathogenesis of the disease is linked to the immune system, but no convincing information about the etiology and in particular about the genetic basis of PFAPA has been obtained so far.

In our clinical practice, we identified a number of families for which PFAPA seems to have a genetic basis. Based on pedigrees analysis, we could infer an autosomal dominant model of inheritance, with a penetrance factor of almost 50%. However, some families showed an apparently dominant inheritance with full penetrance (e.g. family K), whereas for others a recessive status could not be excluded *a priori* (e.g. family F). According to these observations, we selected the seven most informative families of our collection to perform a genome-wide linkage study. This analysis revealed a clear peak on the long arm of chromosome 8, notably between 8q24.1 and 8q24.3. This region contained a small number of genes and was very close to the telomere of chromosome 8. The significance of the identified area was confirmed using non-parametric analysis, and by comparing haplotypes of affected individuals against non-affected ones. Interestingly, the identified region contained several lncRNAs and many miRNAs, as well as other unknown genes or genes without a clear function. Based on these data we therefore performed a custom sequence capture of all known exons for genes in the interval. The custom capture chip was designed based on annotation of build hg35.1 of the human genome. This implies that we could have missed some recently discovered genes, and all the hypothetical genes and lncRNAs that corresponded to the majority of elements annotated in the locus. The sequencing did not reveal any single mutated gene shared by all affected individuals.

To exclude the possibility that we did not detect a causative disease gene because of inconsistencies in the inferred inheritance pattern, we decided to sequence the entire exome of 11 affected individuals, one “obligate carrier” (i.e. a healthy individual connecting two branches of a pedigree with affected subjects), and one unaffected family member. Our analyses revealed the absence of a unique gene that was mutated in all the affected individuals, including genes and noncoding RNAs lying in the critical region on chromosome 8. Although we cannot exclude *a priori* that mutations that escape exome sequencing such as intronic variants and copy number variations could be the cause of the disease, our data disfavor genetic homogeneity for PFAPA.

We then decided to investigate the possibility that mild mutations or rare variants in other known autoinflammatory genes could cause the PFAPA phenotype. This hypothesis is currently a matter of debate, since the diagnosis of milder forms of autoinflammatory disease and PFAPA is often overlapping, leading to diagnostic uncertainties. In particular, low-frequency polymorphisms (common in 1-4% of the healthy population) have been associated with PFAPA or milder forms of autoinflammatory diseases, mostly because no other strong changes in candidate genes were identified^17^,^19^,^30^. Our analysis revealed that, although we identified a few rare missense changes in some inflammatory genes, the frequencies of these variants were not different from those that were present in the general population, indicating that they may not be sufficient to induce the disease. Moreover, some of these changes did not co-segregate with the phenotype, or were also present in healthy members of the family. This was for example observed for the *NLRP3* variant V198M (p.V200M). This change has been reported in several different forms of *NLRP3*-associated syndrome, such as classical CAPS^22^,^23^,^29^, or other autoinflammatory manifestation^24^,^31^, although it is classified as a low penetrant mutation, due to the fact that it is present in a number of healthy carriers (in the Lausanne control population it is present with a frequency of about 1%). The role of this variant is still debated, but it is regrettably reported as a *bona fide* pathogenic mutation in medical genetics diagnostic protocols. The presence of this variant only in the unaffected individuals of family O strongly contradicts its possible role in generating an inflammatory phenotype. A recent paper, probably the most complete clinical study of V198M, showed that 19 cases of CAPS out of 830 carried V198M, although 3 of these harbored other possible candidates in other inflammatory genes, and V198M was present in 7 asymptomatic individuals related to these 19 patients^25^. Furthermore, Valine at codon 200 is not at all conserved through evolution, suggesting that this amino acid residue is likely not detrimental for protein function. Other mammals such as rat, dog and mouse carry a methionine at the same position of their natural NLRP3 orthologues. Altogether, these data indicate that V198M (p.V200M) is likely a neutral human polymorphism and that patients carrying this variant should probably be re-analyzed for other causative mutations.

Another example is the novel change found in patient R1, the NLRP12 p.R211C, which is present in a well-conserved domain and has a predicted strong impact on protein function. Recently a similar missense mutation (p.R352C) in the same NACHT domain of this gene was associated with genetically unexplained periodic fevers^32^, with a PFAPA-like phenotype, and was shown to cause a functional defect in Caspase-1 signaling. The fact that R1’s unaffected sister carries this allele likely indicates that this change has no effects (or has at best a hypomorphic role) at the clinical level. It is interesting to notice that the affected patient (R1) also carried another very rare change in the gene *MEFV* (p.F425Y) with function unknown. The intriguing hypothesis of digenic or multigenic mutations causing the impairment of the inflammasome seemed to be reinforced by the identification of two polymorphisms often associated with the disease in family A. More specifically, we identified a change in the *MEFV* gene (p.E148Q) and in *NLRP3* (p.Q703K), both common polymorphisms with a possible functional effect on the protein. In particular, for p.Q703K, functional impairment of *NLRP3* has been clearly demonstrated^33^. Unfortunately this digenic pattern of inheritance was not identified in other affected subjects within known autoinflammatory-associated genes^34^. Nevertheless we cannot exclude, based on this observation, that other rare variants present in other inflammasome-associated genes could be involved in the etiology of PFAPA.

To investigate this possibility we carefully screened some of the genes that compose the inflammasome set (in particular the NLR family members), based on current literature^35^,^36^,^37^. We identified a few novel variants in *NLRP2*, *NLRP5* and *NOD1*, as well as some very rare variants in other inflammasome-associated genes, such as *NLRP10, NLRP4*, *NLRP11* and *NLRC4*. Interestingly, the majority of these genes were involved in IL-1β inflammasome activation, or in the NF-κB pathway. For example, new evidence demonstrated the high similarity and redundancy across the structure of NLRP1, NLRP6, NLRP10, NLRP3 and NLRP12^38^. Although the function within the inflammasome for many of these members has not yet been clarified, structural similarities among NLRs suggest that all these components could somehow mediate inflammation in response to different stimuli and trigger IL-1β production^37^. Moreover, we identified two samples carrying a specific rare haplotype in *NLRP1* that has recently been associated with vitiligo and autoimmune diseases, and connected to an increase of IL-1β release in patients carrying the haplotype^39^. Dominant mutations in the same gene have been shown to cause systemic inflammation in mouse models^40^. Another observation from the analysis on the possible effect on protein structure was that the majority of identified variants seemed to be present in or in proximity of the NACHT domain. This domain has an ATPase activity and is crucial for the self-oligomerization (usually to form heptamers or hexamers) of NLRs to form the structure necessary for inflammasome assembly^41^. NACHT domain mutations have been associated with structural changes that lead to a continuous activation of the inflammasome complex. A link between mutations affecting NACHT and different autoinflammatory diseases has been observed in *NLRP3* and *NOD1* for example^42^,^43^, reinforcing the hypothesis of a gain-of-function effect of these mutations on the formation of the complex.

It is interesting to observe that every single affected individual in this cohort presented more than one rare variant in one of the inflammasome-composing genes. Unfortunately, due to large number of genes involved, it is impossible to perform a meaningful statistical analysis based on this observation, unless extremely large groups of PFAPA patients are recruited and analyzed, possibly as part of an extended international effort. However, it may not be unreasonable to start thinking of a “total inflammatory burden” similar to the concept of “total ciliary burden” proposed for disease such as the Bardet-Biedl syndrome, that has developed from a simple Mendelian disorder into a trigenic^44^,^45^ and finally into an oligogenic disorder^46^.

In conclusion, the absence of a common gene harboring exonic mutations in all the affected individuals analyzed in this work suggests that PFAPA syndrome is transmitted either as Mendelian disease with high genetic heterogeneity, or as an oligogenic or complex trait. Mutations in noncoding or poorly covered regions of the genome that may have escaped our analysis are also possible, and further studies are needed to assess their potential presence in PFAPA patients. Oligogenic inheritance would be consistent with our observation that affected individuals may harbor multiple variants in inflammasome-associated genes. However, given the fact that such variants may be shared with a significant number of the general unaffected population, specific studies are needed to underpin or refute this hypothesis, and extreme caution should be used prior to indicating causality.

## Methods

### Patient selection and ethical commitment

The families participating in this study were from a large collection of PFAPA patients who were ascertained at the Center for Pediatric Rheumatology of Western Switzerland at the Lausanne University Hospital and the Geneva University Hospital. The familial clustering and inheritance patterns were investigated by administrating a specific survey to family members of the affected patients. The diagnosis of PFAPA in family members was based on the report, by the affected persons or their parents, of a stereotypical and recurrent pattern of illness during their own childhood. The PFAPA phenotype was defined according to previously published clinical criteria^47^; in addition a genetic screening for one or more monogenic periodic fever syndromes (FMF, TRAPS, HIDS) was performed for selected individuals. Our study was designed in accordance with the tenets of the declaration on Helsinki and was approved by the Institutional Review Boards of the University of Lausanne and of the Lausanne University Hospital. Written informed consent was obtained from all patients who enrolled in the study. As controls for the screening of rare variants, data obtained by exome sequencing of 416 healthy anonymous individuals from a large population-based study done in Lausanne (the CoLaus cohort) were used^48^.

### DNA extraction and lymphocytes immortalization

DNA was extracted from peripheral leukocytes (PBLs) of participants by using the NucleonBaCC3 DNA extraction kit (GE healthcare, Life Science). For some family members and for all probands, white mononucleate cells were isolated using Ficoll-Paque PLUS (GE healthcare Life Science), according to the manufacturer’s instruction. Once isolated, cells were processed for EBV immortalization using classical protocols^49^, optimized for our requirements as follows. After three washes with PBS and 2% FCS, 1 × 10^6^ PBL cells were transferred to a 10 ml Falcon tube, diluted in 1 ml of LCC medium (RPMI 1640 medium, 20% serum, 2 μg/ml Cyclosporine A) and incubated with a EBV virus suspension for 1 h. Cells were then plated into single wells from a 12-well plate and kept in culture for ~1 month. Following this immortalization process, cells were frozen in RPMI 1640 with 20% serum and 10% DMSO for cryopreservation.

### Genotyping and linkage analysis

Six out of 14 families were selected for whole genome genotyping, based on the availability of clinical information and the size of the family trees. DNA from all available members of each of these families was assessed by the Illumina Human Linkage12 array (Illumina, CA), containing more than 6000 highly heterozygous SNPs, with an average distance between SNPs of about 441 kb. Output was then analyzed using the Genotype Studio software (Illumina Inc., CA) and the SNPs calls were filtered based on quality and the presence of Mendelian inconsistencies. Genotype data were handled using the commercial software Progeny 7 (Progeny Software LLC) to generate appropriates files for linkage analysis software, carried out with the Merlin package and by using the Vital-IT high performance computer structure (the Swiss Institute of Bioinformatics, Lausanne, www.vital-it.ch). Both parametric and not-parametric analysis models were tested. Because of the lack of data on genetic background for this disease, we could not perform a classical computational approach to estimate the penetrance factor for the parametric analysis. Therefore, penetrance factor for parametric analysis was empirically calculated based on the observed families as a ratio between the number of affected patients and the total number of subjects predicted to be affected based on the model.

### Next generation sequencing

In the first sequencing experiment, aimed at determining DNA variants on chromosome 8 interval, a custom made sequence capture chip, based on Agilent SureSelect technology, was designed by Genotypic (Bangalore, India). Captured DNA was then sequenced by Fasteris (Geneva, Switzerland) by using a single lane of the Illumina Genome Analyzer II (Illumina, San Diego CA).

For exomes, the entire capturing and sequencing procedures were performed at the Lausanne Genomic Facility (GTF). Genomic DNA samples were barcoded and exons were captured using the SureSelect Exome kit V5 (Agilent, Santa Clara, CA) according to the manufacturer’s protocols. Sequencing was performed using again a single lane of the Illumina Genome Analyzer II (Illumina, San Diego, CA). Reads were aligned to the reference sequence (human genome reference 19 – hg19) using Novoalign 2.08 (Novocraft.com). The obtained coverage was 10x or higher for 92% of the targeted regions. Base quality score recalibration, indel realignment, duplicates removal and SNP and INDEL calling were performed by using Genome Analysis Tools kit (GATK)^50^. Variant annotation and filtering was performed with both Annovar^51^ and the Ingenuity Variant Analyzer web interface (Ingenuity System, CA). A subset of the identified variants were confirmed and analyzed by classical Sanger sequencing according standard protocols. Statistical analysis on rare variants present in inflammasome related genes was performed using the Graphpad on-line calculator (http://graphpad.com). A two-tails χ^2^ test adjusted for 2 × 2 contingency table was used.

## Author Contributions

SADG, ASF, MH, CR wrote the manuscript, SADG, NB, AvSG, FV, MH performed data acquisition, SADG, NB, ASF, MH, CR analyzed the data, and SADG, ASF, MH, CR designed the study, All authors reviewed the manuscript.

## Additional Information

**How to cite this article**: Di Gioia, S. A. *et al*. Analysis of the genetic basis of periodic fever with aphthous stomatitis, pharyngitis, and cervical adenitis (PFAPA) syndrome. *Sci. Rep.* **5**, 10200; doi: 10.1038/srep10200 (2015).

## Supplementary Material

Supporting Information

## Figures and Tables

**Table 1:** Clinical characterization of patients analyzed by whole exome sequencing.

Patient*	**Age at onset (months)**	**m/f**	**Major symptoms**	**Other symptoms**	**Positive FH**
A1	54	f	P		Brother A2: PFAPA
A2	60	m	P	AbdP, D	Sister A1: PFAPA
B1	6	f	AD		Cousin: PFAPA
F1	12	m	AD	AbdP, H	
O1	41	f	APH	AbdP	
R1	26	f	P, AD	AR	
PLA 6209	18	f	P, AD, APH		Both parents: TE, Father: recurrent P
PLA 6509	4	m	P, AD	AbdP	Both parents: TE
PLA 5008	16	m	P, AD, APH		Father: TE

FH: family history, P: pharyngitis, AD: adenitis, APH: oral aphtosis, AbdP: abdominal pain, M: myalgia, AR: arthralgia, R: rash, H: headache, D: diarrhea, TE: tonsillectomy.

^*^Individuals O5, and R2 were not affected and thus were not examined. Individual B7 was not available for examination.

**Table 2:** Variant-carrying genes that were common to all affected individuals.

**Gene name**	**Number of variants**	**Cases carrying one or more variants**	**Exclusion criteria**
*WNK1*	54	100%	Alignment mistake
*MUC6*	40	100%	Mucin gene
*MUC2*	19	100%	Mucin gene
*NBPF15*	11	100%	Many novel variants, also present in controls
*ZNF384*	1	100%	Bad reference
*OR4C45*	1	100%	Many novel variants, also present in controls
*MUC17*	91	90%	Mucin gene
*MUC16*	26	90%	Mucin gene
*TTN*	18	90%	Low coverage regions
*ZNF492/ZNF98*	16	90%	Many novel variants, also present in controls
*GOGAL6L1*	14	90%	Not well annotated gene
*PCMTD1*	8	90%	Many novel variants, also present in controls
*C11orf40*	3	90%	Suspected false positive change in dbSNP
*KIAA1751*	2	90%	Suspected false positive change in dbSNP

**Table 3:** Variants in NLR and inflammasome-related genes, and protein domains affected by them.

**GENE**	**rs number**	**Samples**	**AA change**	**MAF**	**Domain**
**IPAF (NLRC4)**	149451729	2941	p.G786V	0.004	LRR6
**NAIP**	61757629	B7	p.A161T	0.02	BIR2
**NLRP1**	61754791	2942	p.V939M	0.02	LLR5
**NLRP2**	142463014	2942	p.S4L	0.01	DAPIN
	novel	B7	p.G504D	0	NACHT
	182098487	2940	p.L607P	0.005	between NACHT and LRR1
**NLRP3**	121908147	O5	p.V198M	0.01	between DAPIN and NACHT
	35829419	A1,A2	p.Q703K	0.05	between NACHT and LRR1
**NLRP4**	117212164	O1,O5	p.T162M	0.004	NACHT
**NLRP5**	novel	F1	p.N255S	0.001	between DAPIN and NACHT
	novel	2940	p.R274Q	0.001	between DAPIN and NACHT
**NLRP10**	150112481	O1	p.I384T	0.001	NACHT
**NLRP11**	11671248	2940	p.S1025L	0.01	C-terminal
**NLRP12**	novel	R1,R2	p.R211C	0.001	NACHT
**NOD1 (CARD4)**	novel	2942	p.C241G	0	NACHT
**NOD3 (NLRC3)**	novel	2940	p.R1005Q	0	LRR15
**NOD4**	16965150	2941, F1	p.S210L	0.03	before NACHT domain
**NOD5 (NLRX1)**	150153921	B7	p.L193V	0.007	NACHT
	145779362	O1,O5	p.R547W	0.007	between NACHT and LRR1
**NWD1**	149694092	F1	p.S306L	0.006	between WD and NACHT

AA: amino acid, MAF: minor allele frequency.
