# Supplementary material for: Analysis of the genetic basis of periodic fever with aphthous stomatitis, pharyngitis, and cervical adenitis (PFAPA) syndrome
Source: Sci Rep. 2015 May 19;5:10200. doi: 10.1038/srep10200 (PMC4437314; doi:10.1038/srep10200)
Supplement: Supporting Information [file srep10200-s1.pdf]

Analysis of the genetic basis of periodic fever with aphthous stomatitis, pharyngitis, and cervical adenitis (PFAPA) syndrome

Silvio Alessandro Di Gioia,[1] Nicola Bedoni,[1] Annette von Scheven-Gête,[2] Federica Vanoni,[2] Andrea Superti-Furga,[3] Michaël Hofer,[2,4*] Carlo Rivolta[1*]

[1]Department of Medical Genetics, University of Lausanne, 1005 Lausanne, Switzerland

[2]Pediatric Rheumatology Unit of Western Switzerland, Department of Pediatrics, CHUV, University Hospital of Lausanne, Lausanne

[3]Division of Molecular Pediatrics Department of Pediatrics, CHUV, University Hospital of Lausanne, Lausanne, Switzerland

[4]Department of Pediatrics, HUG, Geneva, Switzerland

*Equally contributing authors

Supplementary Table 1. List of genes present in the interval (hg17)

Gene	Function	Gene	Function
**FAM84B**	Unknown	**OC90**	Subunit of otoconin
**PCAT1**	lncRNA	**HHLA1**	Human endogenous retrovirus
**POU5F1B**	Probably transcription factor	**KCNQ3**	Potassium channel subunit
**LOC727677**	lncRNA	**HPYR1**	lncRNA
**MYC**	Transcription factot	**LRRC6**	Ciliary protein
**MIR1204**	miRNA	**TMEM71**	Unknown
**PVT1**	lncRNA	**PHF20L1**	Unknown
**MIR1205**	miRNA	**TG**	thyroglobulin
**MIR1206**	miRNA	**SLA**	Regulator of T cells
**MIR1207**	miRNA	**WISP1**	lncRNA
**MIR1208**	miRNA	**NDRG1**	Mitotic spindle protein
**LOC728724**	lncRNA	**ST3GAL1**	Glxcosilating protein
**GSDMC**	Leucin zip. Not known	**ZFAT**	Transcription factot
**FAM49B**	Unknown	**ZFAT-AS1**	lncRNA
**MIR5194**	miRNA	**MIR30B**	miRNA
**ASAP1**	Membrane trafficking	**MIR30D**	miRNA
**LOC100507117**	lncRNA	**LOC286094**	lncRNA
**ASAP1-IT1**	lncRNA	**KHDRBS3**	RNA binding protein
**ADCY8**	Adenilate cyclase brain specific	**FAM135B**	Unknown
**EFR3A**	Membrane protein involved in hearing	**COL22A1**	Collagen protein

Analysis of the genetic basis of periodic fever with aphthous stomatitis, pharyngitis, and cervical adenitis (PFAPA) syndrome. Silvio Alessandro Di Gioia, Nicola Bedoni, Annette von Scheven-Gête, Federica Vanoni, Andrea Superti-Furga, Michaël Hofer, Carlo Rivolta

Supplementary Table 2. Relevant DNA changes on chromosome 8's critical region

Chromosome	Position	Gene Region	Gene Symbol	Protein Variant	Case Samples With Variant	L1_PFAPA - Read Depth	C1 - Read Depth	B1 - Read Depth	Translation Impact	SIFT Function Prediction	dbSNP ID
8	131136174	Intronic	ASAP1		1	10					
8	131199667	Intronic	ASAP1		1	26					374655037
8	131308755	Intronic; ncRNA	ASAP1; ASAP1-IT1		1		29				
8	131859791	Intronic	ADCY8		1			17			
8	132952727	Intronic	EFR3A		1	84					34080810
8	133023148	3'UTR	EFR3A		1		133				
8	133036718	3'UTR	OC90		1	522					11984482
8	133041571	Intronic	OC90		1	263					187677649
8	133044122	Intronic	OC90		1		65				61756131
8	133062088	Intronic	OC90		2	476		12			140801563
8	133074915	3'UTR	HHLA1		1	350					184490995
8	133075629	3'UTR	HHLA1		1	133					189956458
8	133076330	Intronic	HHLA1		1	222					185769537
8	133088451	Intronic	HHLA1		1	90					80015412
8	133088536	Intronic	HHLA1		1	23					117096489
8	133088610	Intronic	HHLA1		1	336					79355606
8	133088884	Intronic	HHLA1		1	277					190888827
8	133146616	Exonic	KCNQ3	p.P454S; p.P574S	1		98		Missense	Tolerated	74582884
8	133200104	Intronic	KCNQ3		2	34	24				112359919
8	133200108	Intronic	KCNQ3		2	32	28				113534923
8	133200146	Intronic	KCNQ3		1	13					
8	133200150	Intronic	KCNQ3		1	14					
8	133200173	Intronic	KCNQ3		1	12					

8	133200175	Intronic	KCNQ3		1	12					
8	133686307	Intronic	LRRC6		1	55					
8	133686319	Intronic	LRRC6		1	69					
8	133686364	Intronic	LRRC6		1			27			
8	133723314	Intronic	TMEM71		1		107				139614933
8	133723332	Intronic	TMEM71		1			53			
8	133860996	3'UTR	PHF20L1		3	401	128	152			200187079
8	133984821	Intronic	TG		3	165	26	57			1554541
8	134052225	Intronic	TG; SLA		1	211					940080
8	134468274	3'UTR	ST3GAL1		1	301					78468413
8	135602612	Intronic	ZFAT		1	50					141077144
8	135610971	Intronic	ZFAT		1	145					117672909
8	139143039	3'UTR	FAM135B		1		109				
8	139218528	Intronic	FAM135B		2	27	23				62530917
8	139219088	Intronic	FAM135B		1	15					147191196
8	139380024	Intronic	FAM135B		1	125					143041039
8	139601612	Exonic	COL22A1	p.P1589T	1	310			Missense	Tolerated	139658465
8	139631866	Intronic	COL22A1		1	376					144729639
8	139668024	Intronic	COL22A1		1	261					117016826
8	139696503	Intronic	COL22A1		1			24			5895552
8	139704646	Intronic	COL22A1		1	11					62527926
8	139704651	Intronic	COL22A1		1	11					62527927
8	139704653	Intronic	COL22A1		1	11					62527928
8	139733184	Intronic	COL22A1		1	225					76691367
8	139774852	Intronic	COL22A1		1	19					
8	139817576	Intronic	COL22A1		1	29					371321111

Supplementary Table 3. Variants present in autoinflammatory and inflammasome related genes, per family

FAMILY A

Gene	rs number	NT change	AA change	MAF	A1	A2	SIFT	Polyphen
**MEFV**	11466045	c.442G>C	p.E148Q	0.007	+/-	+/-	Damaging	Probably damaging
**NLRP3**	35829419	c.2113C>A	p.Q705K	0.04	+/-	+/-	Tolerated	Benign
**CARD8**	2043211	c.304T>A	p.F52I	0.3	+/-	+/-	Damaging	Damaging
	140826611	c.440_441dupAA	p.Val148LysfsX26	0.04	+/+	+/-	Damaging	Damaging

FAMILY B

Gene	rs number	NT change	AA change	MAF	B1	B7	SIFT	Polyphen
**TNFRSF1A**	4149584	c.362G>A	p.R121Q	0.018	+/-	+/-	Tolerated	Probably damaging
**TNFRSF8**	2230624	c.818G>A	p.C273Y	0.013	+/-	+/-	Damaging	Probably damaging
**CARD8**	2043211	c.304T>A	p.F52I	0.3	+/-	+/-	Damaging	Damaging
**NLRP2**	novel	c.1511G>A	p.G504D	-	+/+	+/-	Tolerated	Probably damaging
**NAIP**	61757629	c.481G>A	p.A161T	0.03	+/+	+/-	Tolerated	Probably damaging
**NOD1**	150153921	c.577C>G	p.L193V	0.007	+/+	+/-	Damaging	Probably damaging

FAMILY O

Gene	rs number	NT change	AA change	MAF	O1	O5	SIFT	Polyphen
**NLRP4**	117212164	c.485C>T	p.T162M	0.036	+/-	+/-	Damaging	Damaging
**NLRP10**	150112481	c.1151T>C	p.I384T	0	+/-	+/+	Damaging	Damaging
**NOD5**	145779362	c.1639C>T	p.R547W	0.007	+/-	+/-	Damaging	Probably damaging
**NLRP3**	121908147	c.592G>A	p.V200M	0.007	+/+	+/-	Tolerated	Benign

Analysis of the genetic basis of periodic fever with aphthous stomatitis, pharyngitis, and cervical adenitis (PFAPA) syndrome. Silvio Alessandro Di Gioia, Nicola Bedoni, Annette von Scheven-Gête, Federica Vanoni, Andrea Superti-Furga, Michaël Hofer, Carlo Rivolta

FAMILY R

Gene	rs number	NT change	AA change	MAF	R1	R2	SIFT	Polyphen
**NLRP12**	NOVEL		p.R211C	.	+/-	+/-	Damaging	Damaging
**MEFV**	104895169	c.1274A>T; c.1760-28T>A	p.F425Y	0.0012	+/-	+/+	Tolerated	Probably damaging
**LPIN3**	NOVEL	c.1168G>T	p.D390Y	.	+/-	+/+	Damaging	Probably damaging
	201671223	c.1807C>T	p.R603C	0.001	+/-	+/-	Tolerated	Bening

Patient F

Gene	rs number	NT change	AA change	MAF	F1	SIFT	Polyphen
**NLRP5**	NOVEL	c.764A>G	p.N255S	-	+/-	Tolerated	Benign
**NOD4**	16965150	c.629C>T	p.S210L	0.03	+/-	Tolerated	Benign
**NWD1**	149694092	c.917C>T	p.S305L	0.007	+/-	Tolerated	Benign

Patient 2940

Gene	rs number	NT change	AA change	MAF	2940	SIFT	Polyphen
**NLRP2**	182098487	c.1889T>C	p.L608P	0.005	+/-	Damag.	Damaging
**NLRP5**	NOVEL	c.821G>A	p.R274Q	.	+/-	Activate	Benign
**NLRP11**	11671248	c.3074C>T	p.S1025L	0.01	+/-	Damag.	Benign
**NOD3**	NOVEL	c.3014G>A	p.R1005Q	.	+/-	Tolerated	-

Patient 2941

Gene	rs number	NT change	AA change	MAF	2941	SIFT	Polyphen
**IPAF**	149451729	c.2357G>T	p.G786V	0.004	+/-	Tolerated	Possibly damaging
**NOD4**	16965150	c.629C>T	p.S210L	0.03	+/-	Tolerated	Benign
**NLRP1**	35596958	c.3265A>G	p.M1089V	0.05	+/-	Activate	Benign
	34733791	c.2994C>T	p.T965I	0.05	+/-	Tolerated	Benign
	11657747	c.2633C>T	p.T878M	0.05	+/-	Tolerated	Benign

Analysis of the genetic basis of periodic fever with aphthous stomatitis, pharyngitis, and cervical adenitis (PFAPA) syndrome. Silvio Alessandro Di Gioia, Nicola Bedoni, Annette von Scheven-Gête, Federica Vanoni, Andrea Superti-Furga, Michaël Hofer, Carlo Rivolta

	52795654	c.2345C>G	p.T782S	0.05	+/-	Tolerated	Benign
	11651595	c.737C>G	p.T246S	0.05	+/-	Tolerated	Benign
**CARD8**	140826611	c.440_441dupAA	p.Val148LysfsX26	0.04	+/+	Damag.	Damaging

Patient 2942

**Gene**	**rs number**	**NT change**	**AA change**	**MAF**	**2942**	**SIFT**	**Polyphen**
**NLRP1**	61754791	c.2815G>A	p.V939M	0.02	+/-	Tolerated	Possibly damaging
**NLRP2**	142463014	c.11C>T	p.S4L	0.01	+		
**NOD1**	NOVEL	c.721T>G	p.C241G	.	+/-	Tolerated	Benign
**CARD8**	140826611	c.440_441dupAA	p.Val148LysfsX26	0.04	+/+	Damag.	Damaging
